# Pyrrolidinyl caffeamide against ischemia/reperfusion injury in cardiomyocytes through AMPK/AKT pathways

**DOI:** 10.1186/s12929-015-0125-3

**Published:** 2015-03-18

**Authors:** Shih-Yi Lee, Hui-Chun Ku, Yueh-Hsiung Kuo, His-Lin Chiu, Ming-Jai Su

**Affiliations:** Institute of Pharmacology, College of Medicine, National Taiwan University, No.1, Sec.1, Jen-Ai Road, Taipei, 100 Taiwan; Division of Pulmonary and Critical Care Medicine, Mackay Memorial Hospital, Taipei, Taiwan; Department of Chinese Pharmaceutical Sciences and Chinese Medicine Resources, China Medical University, Taichung, Taiwan; Department of Biotechnology, Asia University, Taichung, Taiwan; Department of Chemistry, National Taiwan University, Taipei, Taiwan

**Keywords:** Caffeic acid, Cardiomyocyte, AKT, AMPK, GLUT4, Metformin, Hypoxia/reoxygenation, Ischemia/reperfusion

## Abstract

**Background:**

Coronary heart disease is a leading cause of death in the world and therapy to reduce injury is still needed. The uncoupling of glycolysis and glucose oxidation induces lactate accumulation during myocardial ischemia/reperfusion (I/R) injury. Cell death occurs and finally leads to myocardial infarction. Caffeic acid, one of the major phenolic constituents in nature, acts as an antioxidant. Pyrrolidinyl caffeamide (PLCA), a new derivative of caffeic acid, was synthesized by our team. We aimed to investigate the effect of PLCA on hypoxia/reoxygenation (H/R) in neonatal rat ventricular myocytes (NRVM) and on myocardial I/R in rats.

**Results:**

Cardiomyocytes were isolated and subjected to 6 h hypoxia followed by 18 h reperfusion. PLCA (0.1 to 3 μM) and metformin (30 μM) were added before hypoxia was initiated. PLCA at 1 μM and metformin at 30 μM exerted similar effects on the improvement of cell viability and the alleviation of cell apoptosis in NRVM after H/R. PLCA promoted p-AMPK, p-AKT, and GLUT4 upregulation to induce a cardioprotective effect in both cell and animal model. The accumulation of cardiac lactate was attenuated by PLCA during myocardial I/R, and infarct size was smaller in rats treated with PLCA (1 mg/kg) than in those treated with caffeic acid (1 mg/kg).

**Conclusions:**

AMPK and AKT are synergistically activated by PLCA, which lead facilities glucose utilization, thereby attenuating lactate accumulation and cell death. The cardioprotective dose of PLCA was lower than those of metformin and caffeic acid. We provide a new insight into this potential drug for the treatment of myocardial I/R injury.

## Background

Coronary heart disease is a leading cause of death in the world [1]. With ischemia in coronary heart disease, impairment of the oxygen supply and metabolic disorder both occur [2]. Without oxygen, anaerobic glycolysis occurs accompanied by lactate accumulation, leading to intracellular acidosis [2]. The PH value fall results in elevating of NADH/NAD^+^ ratio and further inhibiting ATP production [3,4]. The re-establishing of blood flow to an ischemic zone is called reperfusion [1]. A high intracellular calcium concentration and the production excess reactive oxygen species inhibit the mitochondrial electron transport chain causing cell damage [5]. Cell apoptosis occurs, such as by activation of caspase-3 activity, and finally leads to myocardial infarction [1]. Reducing the size of myocardial infarct is the determining factor of clinical outcomes in acute coronary artery disease [6,7]. A therapeutic drug that targets ischemia reperfusion (I/R) injury is needed and has yet to be developed.

AMPK, an important energy sensor and metabolic regulator, is modulated by the ratio of [ATP]/[AMP] × [ADP] [8]. This is important in the setting of myocardial ischemia reperfusion (I/R) due to high energy demands and low energy reserves. During hypoxia, the decline of ATP induces AMPK activation [9]. AMPK phosphorylation enhances glycolysis by two mechanisms. In the first, glucose uptake is increased by stimulation of GLUT4 expression [10]. In the second, 6-phosphofructo-2-kinase activity is enhanced [11]. AMPK also increases fatty acid oxidation by phosphorylating and inactivating acetyl-CoA carboxylase, along with the decreasing concentration of malonyl-CoA, an inhibitor of fatty acid transport into mitochondria [12]. Metformin, an anti-hyperglycemic agent that activates AMPK [13], is known to have cardioprotective effects against I/R injury [14].

Energy production is also controlled by AKT, which is a serine/threonine protein kinase [15]. PI3K/AKT signaling is involved in the insulin pathway, which plays a key role on glucose metabolism [16]. AKT phosphorylation increases glucose utilization by increasing glycolysis and glucose oxidation [16-18]. AKT enhances the expression and translocation of GLUT4 through the phosphorylation of AS160 to promotes glucose uptake [16,19], and increases glycolysis by phosphorylating hexokinase [17]. AKT inhibits fatty acid oxidation through down regulation of PPARα/PCG-1-dependent transcription [18] and promote glucose oxidation via Randle cycle mechanism [20]. Facilitation of glucose utilization contributes to the protective effect of AKT signaling to reduce infarct size and improve myocardial function in a heart subjected to I/R [15].

**Figure 1:**
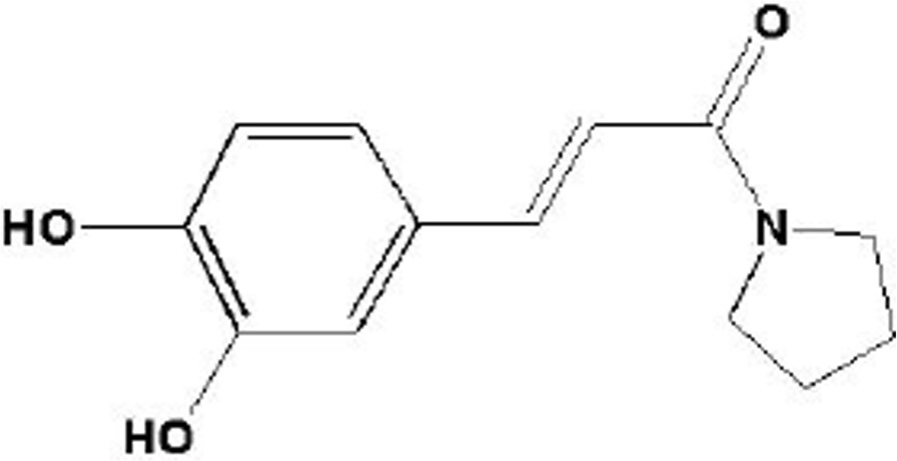
**Structure of pyrrolidinyl caffeamide (PLCA).**

Caffeic acid, one of the major phenolic constituents in nature, acts as an antioxidant [21]. Previous research has revealed that caffeic acid reduces oxidative stress and exerts a protective effect on the cardiovascular system [22,23]. In addition, caffeic acid has been reported to activate AMPK [24]. Our new synthetic derivative of caffeic acid is pyrrolidinyl caffeamide (PLCA) from the laboratory of YH Kao’s (Figure 1). The effect of PLCA on the cardiovascular system is unknown; therefore, we aimed to assess the protective effects of PLCA on hypoxia/reoxygenation (H/R) induced in neonatal ventricular myocytes and myocardial I/R injury in rats.

## Methods

### Isolated neonatal rat ventricle myocytes (NRVM)

The investigation conformed to the Guide for the Care and Use of Laboratory Animals published by the US National Institutes of Health (NIH Publication No. 85–23, revised 1996). NRVM was isolated from 2-day-old Sprague–Dawley rats. Hearts were excised and digested with enzyme mixtures containing pancreatin (Sigma-Aldrich, St. Louis, MO, USA) and collagenase (GIBCO, Grand Island, NY, USA) dissolved in Ca^2+^/Mg^2+^-free Hank’s balanced saline solution. Cell suspensions were pre-plated for 90 min by seeding on an uncoated plate to select the cardiac fibroblasts. The unattached cells were NRVM, which were cultured with Eagle’s minimal essential medium supplemented with 10% fetal calf serum and antibiotics (100 μg/ml penicillin and 100 μg/ml streptomycin) at 37°C under a 5% CO_2_ − 95% air atmosphere for the first day. On the second day, a confluent monolayer of spontaneously beating NRVMs was formed. All experiments were performed with contractile myocyte-rich monolayers with 90-95% confluence. Serum was deprived for 12 h before experiments initiated.

### Experimental model of H/R in NRVM

NRVM were exposed to 6 h hypoxia (5% CO_2_, 1.8% O_2_, 93.2% N_2_) followed by 12 h reoxygenation. The H/R model mimics I/R injury in clinical conditions. In the normoxia condition, 2.2 g/L NaHCO_3_ was added to the medium to adjust PH to 7.4. In the hypoxia condition, sodium lactate (8 μM) was added to the medium to induce stress, and the PH value of the medium was adjusted to 6.8 by adding 0.57 g/L NaHCO_3_, according to the Henderson-Hasselbalch equation: PH = 6.1 + log [52 × (mg/ml NaHCO_3_/%CO_2_) -1] [25]. PLCA (0.1, 0.3, 1, and 3 μM) and metformin (30 μM) were added to the medium before hypoxia was initiated. Compound C (15 μM) and LY294002 (15 μM) were added to evaluate the mechanisms of PLCA.

### Detection of cell viability

Cell viability was determined by MTT assay as describe prevsiouly [26]. Cells were treated with MTT (3-(4,5-Dimethylthiazol-2-yl)-2,5-diphenyltetrazoliumbromide) at 0.5 mg/ml. The purple formazan crystals were dissolved in DMSO. Solutions were then loaded in a 96 well plate, and determined on an automated microplate spectrophotometer at 570 nm. Each condition tested was performed in triplicate in each experiment.

### Detection of cell death

Cell death was detected with a detection kit (BD, USA). Cells were incubated with Annexin V-FITC and propidium iodide (PI). The reagent was incubated at room temperature for 15 min in the dark, and detected by fluorescence microscopy. In this procedure, green fluorescence indicates early apoptosis (Annexin V-FITC positive and PI negative) and orange indicates late apoptosis or death (annexin V-FITC positive and PI positive).

### Experimental model of myocardial I/R injury in vivo

Eight-week-old male Sprague–Dawley rats (BioLASCO Taiwan, Co., Ltd, Taipei, Taiwan) were used. Animals were maintained under a 12-h light/dark cycle at a controlled temperature (21 ± 2°C) with free access to food and tap water. Rats were anesthetized and subjected to 1 h coronary artery occlusion, followed by 2 h reperfusion as described previously [27]. PLCA (1 mg/kg) or caffeic acid (1 mg/ kg) was administered by intraperitoneal injection 15 min before I/R started. At the end of I/R, the hearts were harvested and the coronary artery was ligated again and perfused with 1% evan blue to distinguish between the normoxia and risk area. Each heart was then sliced horizontally, incubated in 1% triphenyltetrazolium chloride (Sigma, St. Louis, MO, USA) for 30 min at 37°C, and then placed in 10% formaldehyde. The infarct area appeared white.

### Protein extraction from cardiac tissue

NRVMs or heart tissue were obtained at the end-point of the experiment. Proteins were homogenized in RIPA buffer (Tris–HCl 50 mM, NaCl 150 mM, EGTA 1 mM, EDTA 1 mM, NP-40 1%, sodium deoxycholate 1%) containing cocktail protease and phosphatase inhibitor (Sigma, St. Louis, MO, USA). The supernatant of homogenate was collected after centrifugation (800 × g, 10 min at 4°C). Protein concentrations were determined by BCA protein assay kit (Thermo Fisher Scientific Inc., Rockford, IL, USA).

### Determination of cardiac lactate level

Heart protein was collected at the end of I/R experiment. Cardiac lactate level was measured by ELISA kit (Biovision, USA).

### Western blot analysis

The protein expression in NRVM or rat heart were analyzed by western blotting with method described previously [26]. Briefly, equal amounts of protein samples were separated by SDS-PAGE and transferred to polyvinylidene difluoride membranes (Perkin-Elmer Life Sciences, Boston, MA, USA). The membranes were blocked in 5% fat-free milk dissolved in TBST (Tris/phosphate/saline/Tween) and incubated with primary antibody to p-AMPK, p-AKT, caspase-3 (Cell Signalling, Beverly, MA, USA), GLUT4 (Santa Cruz Biotechnology, Santa Cruz, CA, USA), and β-actin (Sigma-Aldrich).

### Statistical analysis

All values are presented as means ± SE. The results were analyzed using ANOVA followed by Bonferroni’s post hoc tests. Significance was set at *p* < 0.05.

## Results

### Comparison of the protective effects of PLCA and metformin in NRVM after H/R stress

**Figure 2:**
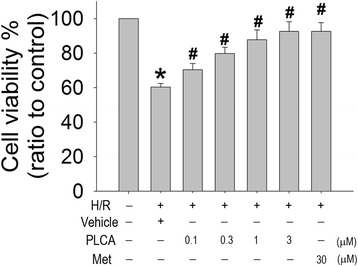
**Effect of PLCA and metformin on cell viability in NRVM after H/R stress.** Cell viability was measured after NRVM subjected to 6 h hypoxia, and followed by 12 h reoxygenation. PLCA (0.1, 0.3, 1, and 3 μM) and metformin (30 μM) were treated before hypoxia initiated. (n = 8) *P < 0.05 vs. control, #P < 0.05 vs. H/R + vehicle. Met = metformin.

To determine whether PLCA exerted a protective effect during the H/R condition, cell viability was assessed via MTT assay. Metformin (30 μM) was used as an active drug for comparison with PLCA. Cell viability declined significantly after H/R stress. PLCA and metformin both improved cell viability in NRVM after H/R stress (Figure 2). PLCA exerted a protective effect that was concentration dependent, ranging from 0.1 to 3 μM. The protective effect of metformin (30 μM) was no greater than that of PLCA (3 μM) in the H/R condition.

### Comparison of the protein expression between PLCA and metformin in NRVM after H/R stress

**Figure 3:**
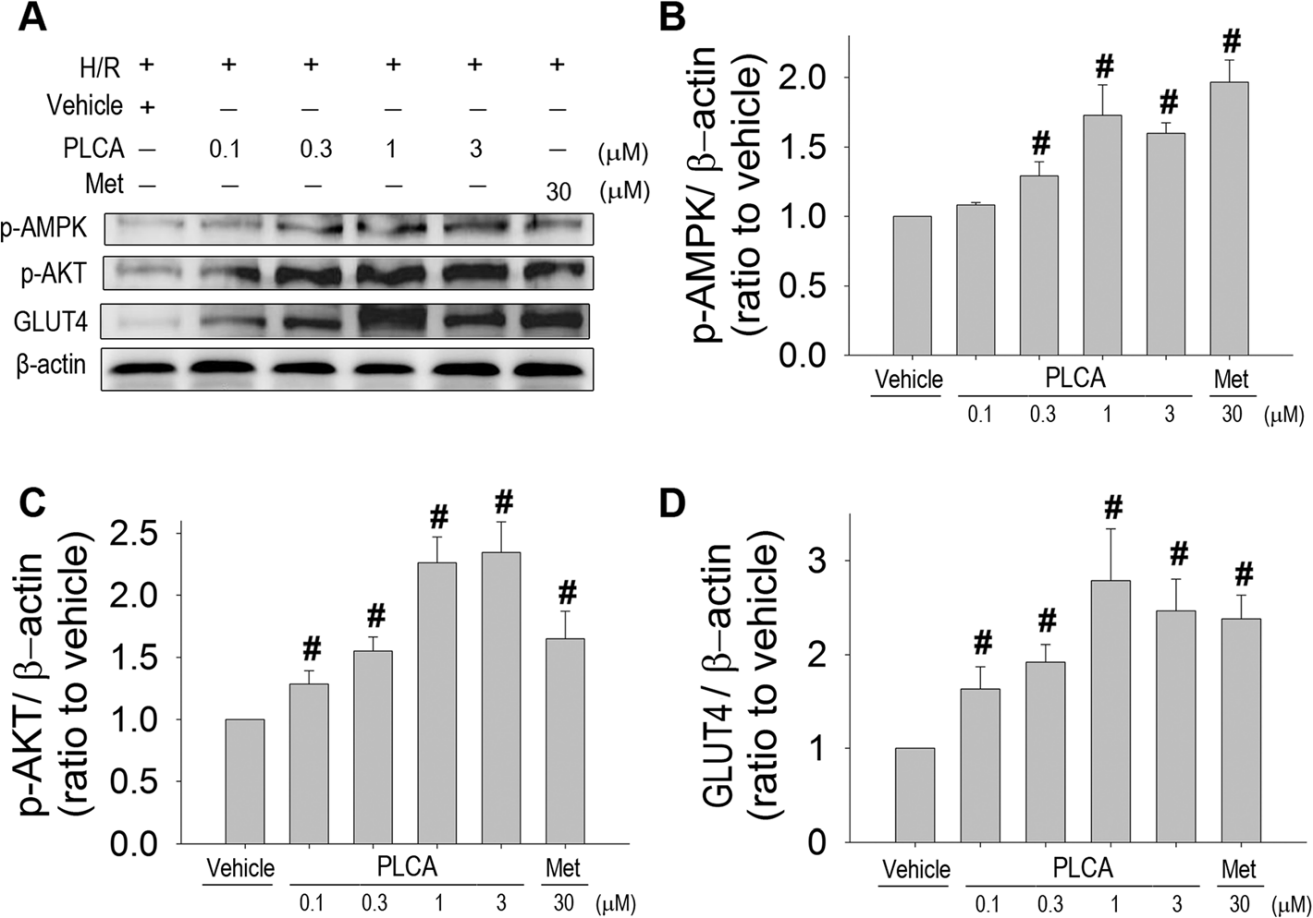
**Effect of PLCA and metformin on protein expression in NRVM after H/R stress.** Protein expression was measured in NRVM after H/R stress. **(A)** Original western blots were shown. Ratios of **(B)** p-AMPK to actin, **(C)** p-AKT to actin, and **(D)** GLUT4 to actin. (n = 8) #P < 0.05 vs. H/R + vehicle. Met = metformin.

To investigate the mechanisms of the protective effects of PLCA and metformin in NRVM during in the H/R condition, we evaluated the expression of several proteins. PLCA and metformin both induced phosphorylation of AMPK (Figure 3A,B) and AKT (Figure 3A,C) in NRVM after H/R stress, along with the elevation of GLUT4 expression (Figure 3A,D). PLCA increased p-AKT, p-AMPK, and GLUT4 expression with concentration dependence, ranging from 0.1 to 1 μM, but did not show further increases of protein expression at 3 μM, indicating a ceiling effect of PLCA at 1 μM. In addition, no significant differences between PLCA (1 μM) and metformin (30 μM) treatment were found in the expression of p-AKT, p-AMPK, and GLUT4 in NRVM after H/R stress.

### PLCA and metformin both alleviated cell death in NRVM after H/R stress

**Figure 4:**
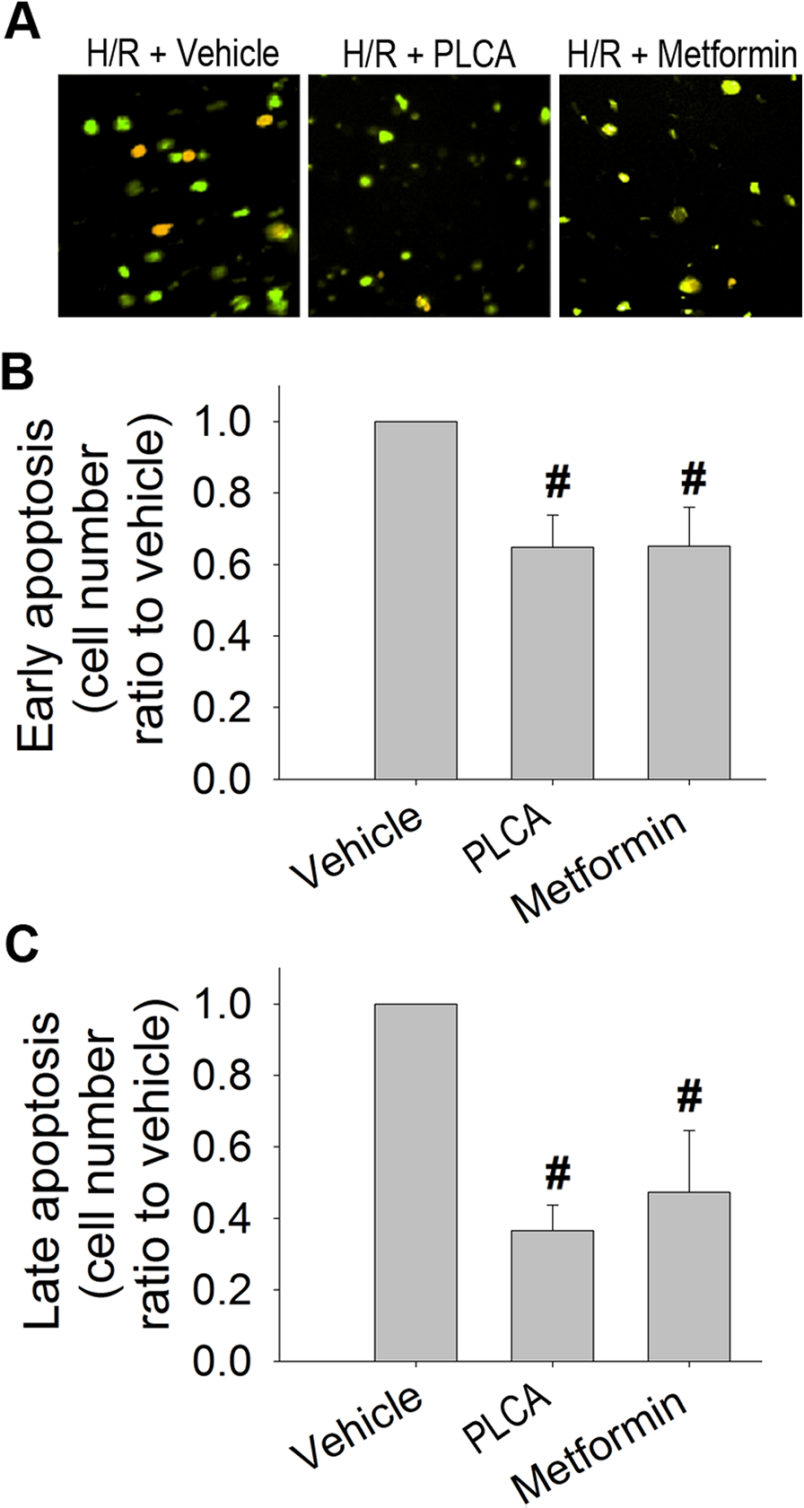
**Effect of PLCA and metformin on cell death in NRVM after H/R stress.** Progression of cell death was measured in NRVM after H/R stress. **(A)** Original microscopy photos were reported. Cells indicated green are in early apoptosis (Annexin V-FITC positive and PI negative). Cells indicated orange are in late apoptosis or already dead (annexin V-FITC positive and PI positive). **(B)** Ratio of early apoptosis cell. **(C)** Ratio of late apoptosis cell. (n = 5) #P < 0.05 vs. H/R + vehicle.

We examined the effect of PLCA (1 μM) and metformin (30 μM) on the progression of cell death in NRVM after H/R stress using Annexin V-FITC and PI staining. Cells shown in green were in an early stage of apoptosis (Figure 4A,B), and those in orange were in a late stage of cell death (Figure 4A,C). It was observed that both PLCA and metformin significantly decreased the numbers of green and orange labeled cells, indicating the alleviation of the progression of cell death in NRVM in the H/R condition. The efficacies of PLCA (1 μM) and metformin (30 μM) were exactly the same. The subsequent experiments only investigate the effect of PLCA (1 μM) in NRVM during H/R condition.

### PLCA exerted a protective effect through the AMPK and AKT pathway in NRVM after H/R stress

**Figure 5:**
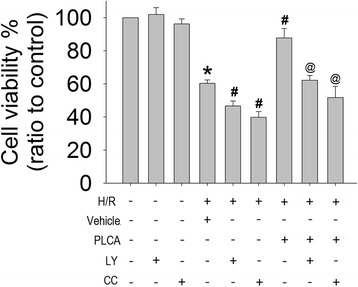
**Effect of the combined treatment of PLCA with compound c or LY294002 on cell viability in NRVM after H/R stress.** Cell viability was measured after NRVM subjected to 6 h hypoxia, and followed by 12 h reoxygenation. Compound C (15 μM) or LY294002 (15 μM) were combined with PLCA (1 μM) before hypoxia initiated. (n = 8) *P < 0.05 vs. control, #P < 0.05 vs. H/R + vehicle, @P < 0.05 vs. H/R + PLCA. Met = metformin; LY = LY294002; CC = compound C.

Since PLCA induced AMPK and AKT phosphorylation in NRVM in the H/R condition, compound C and LY294002 were used to block the AMPK and AKT pathways, respectively, in order to confirm the contributions of AMPK and AKT signaling to the protective effects. Compound C or LY294002 alone did not affect the cell viability in the normoxia condition, but cell viability was decreased in the H/R condition (Figure 5), indicating that AMPK and AKT play important roles during H/R. When PLCA combined with compound C or LY294002, PLCA lost its protective effect on cell viability in NRVM after H/R stress.

**Figure 6:**
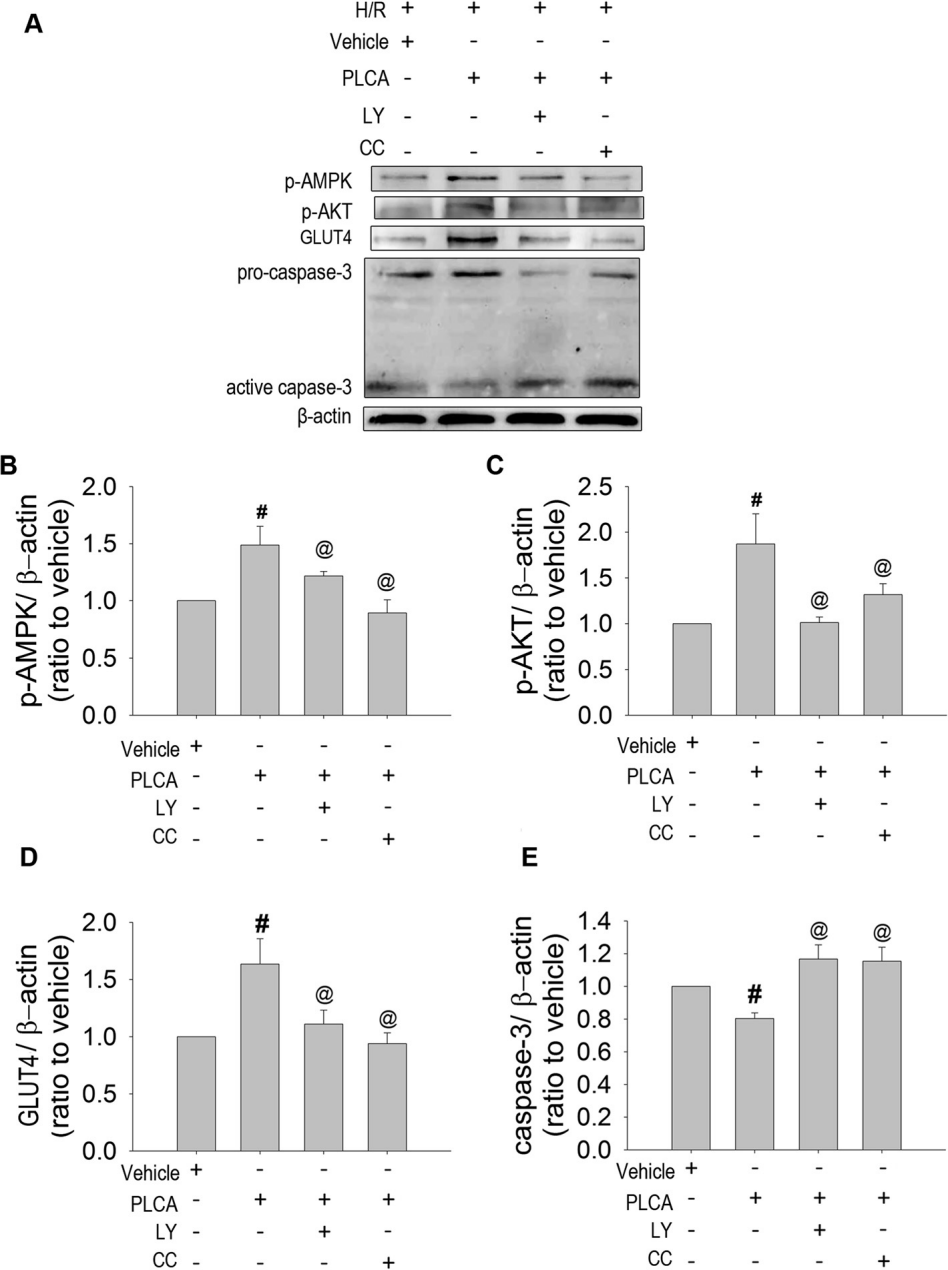
**Effect of the combined treatment of PLCA with compound c or LY294002 on protein expression in NRVM after H/R stress.** Protein expression was measured in NRVM after H/R stress. **(A)** Original western blots were shown. Ratios of **(B)** p-AMPK to actin, **(C)** p-AKT to actin, **(D)** GLUT4 to actin, and **(E)** caspase-3 to actin. (n = 8) #P < 0.05 vs. H/R+ vehicle, @ < 0.05 vs. H/R + PLCA. Met = metformin; LY = LY294002; CC = compound C.

We then investigated the protein expression of PLCA treatment combined with compound C or LY294002. The elevation of p-AMPK and p-AKT expression by PLCA in NRVM in the H/R condition was completely abrogated by its inhibitor, compound C or LY29400, respectively (Figure 6A,B,C). Interestingly, LY29400 decreased the enhanced expression of p-AMPK in PLCA- treated NRVMs, while compound C also diminished the enhanced expression of p-AKT expression in PLCA- treated NRVMs, indicating cross-talk between AKT and AMPK signaling. In addition, the elevation of GLUT4 expression by PLCA in NRVMs during H/R condition was completely abolished by compound C and LY29400 (Figure 6A,D). Active caspase-3 expression was reduced by PLCA in NRVM after H/R stress, and the protective effect was abrogated by compound C and LY29400 (Figure 6A,E).

### PLCA alleviated injury in rats subjected to myocardial I/R

**Figure 7:**
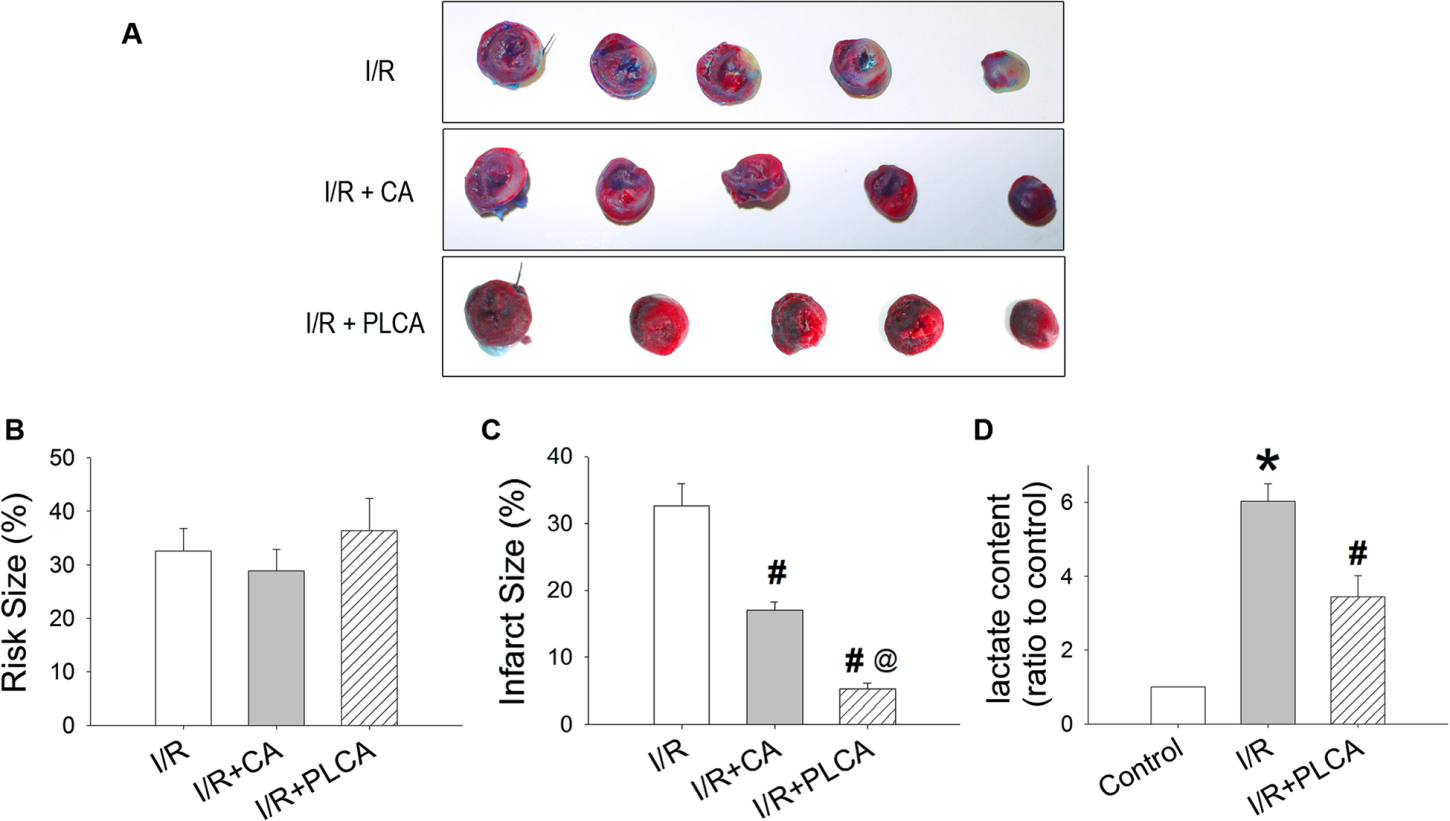
**Effect of PLCA on myocardial I/R induced injury.** Cardiac injury was shown after rats subjected to 1 h coronary artery occlusion and followed by 2 h reperfusion. **(A)** Representative cardiac slices after I/R were shown. **(B)** Risk size and **(C)** infarct size were reported. The non-ischemic myocardium stained blue, and the remnants indicated ischemic myocardium. The infarct myocardium stained white, and the non-infarct myocardium stained red. Ratio of red plus white zone to total zone was defined as risk size. Ratio of white zone to red plus white zone was defined as infarct size. **(D)** Cardiac lactate level was measured. (n = 6) *P < 0.05 vs. control, #P < 0.05 vs. I/R, @ < 0.05 vs. I/R + CA. CA = caffeic acid.

The risk size was similar among groups (Figure 7A and B), indicating that the procedure and severity of coronary artery occlusion did not differ significantly among groups. Rats subjected to coronary artery occlusion for 1 h followed by reperfusion for 2 h developed myocardial infarction (Figure 7A and C). The protective effects of caffeic acid and PLCA were compared. PLCA was shown to attenuate infarct size more than caffeic acid did, by decreasing 47.9% and 84.1% of the I/R group, respectively, at 1 mg/kg. The elevation of cardiac lactate concentration after I/R injury was ameliorated by PLCA (Figure 7D).

### PLCA improved p-AMPK, p-AKT, and GLUT4 expression in rats subjected to myocardial I/R

**Figure 8:**
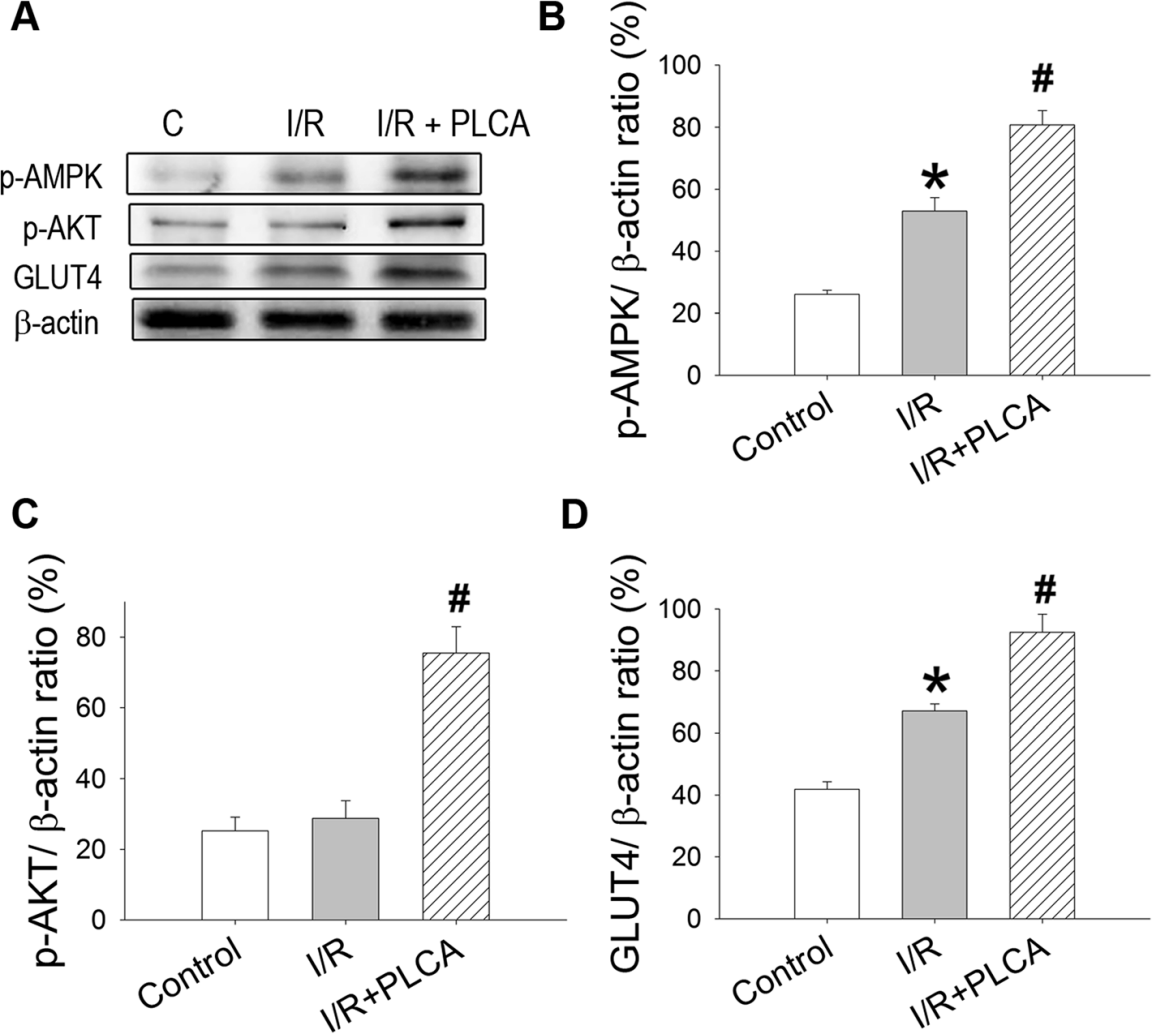
**Effect of PLCA on protein expression in heart after I/R stress.** Protein expression was measured in heart after I/R stress. **(A)** Original western blots were shown. Ratios of **(B)** p-AMPK to actin, **(C)** p-AKT to actin, and **(D)** GLUT4 to actin. (n = 4) *P < 0.05 vs. control, #P < 0.05 vs. I/R.

I/R resulted in 2.03-fold elevation of p-AMPK expression, along with 1.06-fold elevation of GLUT4 expression (Figure 8), but did not affect p-AKT expression. Treatment with PLCA during I/R induced further prominent elevation of p-AMPK, GLUT4, and p-AKT expression, being 1.53-, 2.62-, and 1.32- folds of I/R, respectively.

## Discussion and conclusions

We demonstrated that both PLCA and metformin improved cell viability and alleviated cell death in NRVM after H/R stress. The protective effect is associated with the elevation of AMPK and AKT phosphorylation, and consequent regulation of downstream proteins, including the induction of GLUT4 expression and the suppression of caspase-3 expression. Treatment with PLCA (1 μM) and metformin (30 μM) induced the same response in regulation of and preservation of NRVM viability in the H/R condition, suggesting that PLCA is more potent than metformin in protecting against H/R injury. In the animal study, PLCA demonstrated higher efficiency in protection against myocardial I/R injury than its original compound, caffeic acid. PLCA is a new potential drug for the treatment of myocardial infarction.

Fatty acids generate more ATP per molecule of substrate than glucose; therefore, the heart predominantly uses fatty acids as fuel in the normal condition [28]. When oxygen supply limits, glucose provides more energy per amount of oxygen than fatty acids [29]. A shift in the myocardial metabolism from fatty acid to glucose during myocardial I/R is more oxygen efficient and prevents deleterious effects [28]. Glucose enters cardiac myocytes via glucose transporters, which is an important determinant for glycolytic flux in cardiomyocyte [30]. GLUT4, the most abundant transporter, translocates to the plasma membrane in response to hypoxia [31]. Expression of GLUT4 is crucial for heart during H/R, while cardiac selective ablation of GLUT4 directly influence cardiac function [32]. In addition, ischemic preconditioning significantly improved cardiac GLUT4 expression in heart, and GLUT4 siRNA blocks the cardioprotective effect on ischemic preconditioning [32,33]. Activation of both AMPK and AKT is involved in glycolysis improvement during heart ischemia [9]. Augmentation of glycolysis becomes a major mechanism for the heart to maintain ATP concentrations in response to the impairment of oxidative phosphorylation [29].

During myocardial reperfusion, fatty acid oxidation quickly recovers and becomes the major source of energy [34]. The high rate of fatty acid oxidation inhibits glucose oxidation through Randle cycle mechanisms [20], resulting in a significant uncoupling between glycolysis and glucose oxidation [34,35]. The uncoupling of glucose metabolism produce lactate, which is a potential source of H^+^ production during reperfusion [2]. Stimulation of fatty acid oxidation by AMPK may lead to uncoupling of glycolysis and glucose oxidation [36]. AMPK activation accompanied by AKT activation may facilitate glucose utilization and decrease the uncoupling of glycolysis and glucose oxidation, which decrease lactate accumulation. Our study demonstrated that PLCA activates both AMPK and AKT to modulate the metabolic pathways in a heart undergoing ischemia/reperfusion. Synergistic activation of AMPK and AKT has been shown to regulate glucose metabolism [37], implying the existence of cross-talk between AMPK and AKT as we found here. We demonstrated that the activations of AKT and AMPK are co-related, since inhibiting one of the activities affects the phosphorylation state of the other. AMPK may regulate AKT phosphorylation [33,38,39], and mTORC2 may be a mediator for AMPK to activate AKT [40]. Inhibition of mTORC1 by AMPK could increase the availability of mTOR to the mTORC2 complex, while mTORC2 directly phosphorylates Akt at Ser473 [38,41]. The cross-talk between AMPK and AKT is complicated. AKT has also been shown to regulate AMPK phosphorylation, since wortmannin, a PI3k inhibitor, blocks AKT and AMPK phosphorylation as well as GLUT4 translocation and subsequent ATP synthesis in ischemic preconditioning [33]. The present study demonstrates that PLCA induces AMPK and AKT in a cooperative manner to benefit the heart by increasing glucose utilization during H/R stress.

Inhibition of apoptosis could decrease the infarct size after I/R [42]. The reperfusion injury salvage kinase pathway against cell apoptosis is through PI3K-AKTcascades [15]. AKT is as a key regulator of metabolism and cell survival [15]. Inhibition of AKT signaling by an inhibitor or genetic deletion abrogates the protective effect, while overexpression of AKT protects cardiomyocytes from apoptosis induced by H/R [43]. Activation of AKT inhibits pro-apoptotic proteins, such as bax, bad, bim, and p53, and consequently inhibits caspase activation, finally exerting a protective effect against myocardial I/R injury [15]. In addition, AMPK activation is also reported to have anti-apoptotic effects via increasing bcl2 expression and decreasing caspase-3 activity, whereas abrogation of AMPK activation results in increasing apoptosis in cardiomyocytes [44,45]. The inhibition of the cell death pathway contributes to the protective action of PLCA during I/R induced cell death.

**Figure 9:**
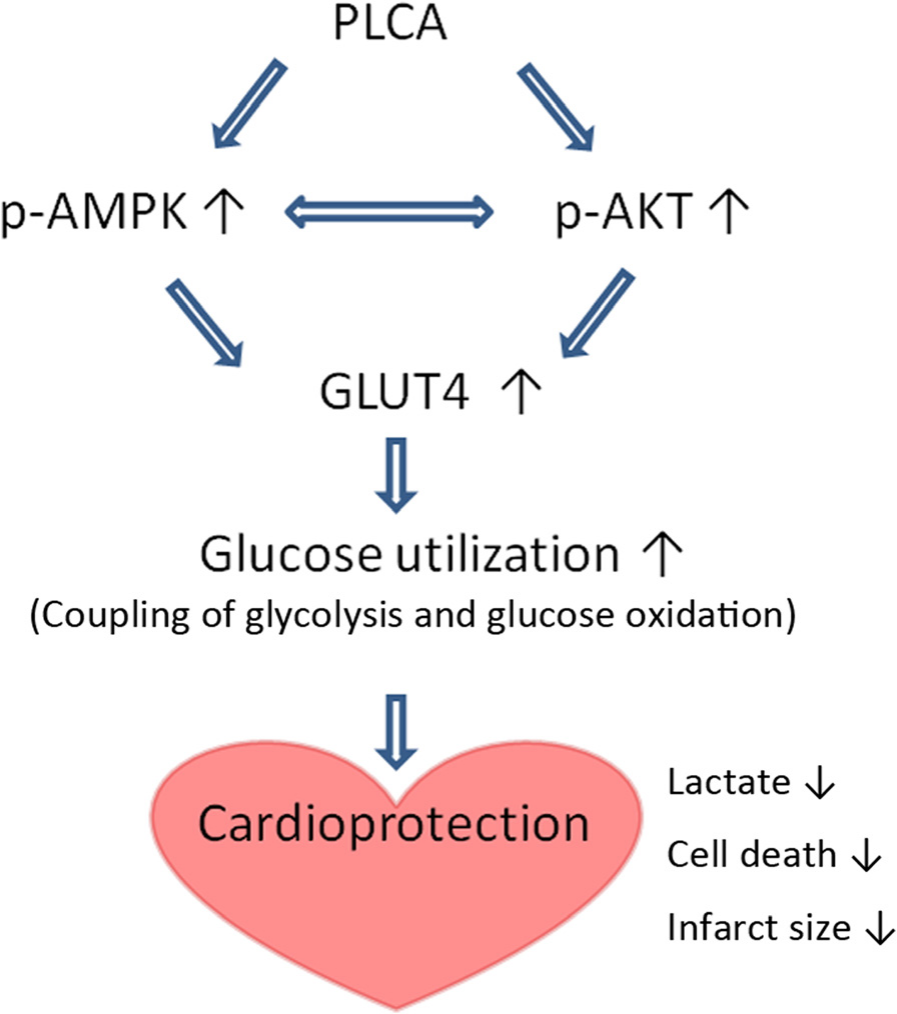
**The mechanisms of PLCA in heart.**

In conclusion, the present study has identified the cardioprotective mechanisms of PLCA. PLCA improves cell viability in NRVM against H/R injury and decrease myocardial infarction in rat against I/R injury by activating AKT- and AMPK-GLUT4 signaling, improving glucose utilization, and decreasing lactate accumulation (Figure 9). We provide a new insight into this potential drug for the treatment of myocardial I/R injury.

## Abbreviations

PLCA: Pyrrolidinyl caffeamide
I/R: Ischemia/reperfusion
H/R: Hypoxia/reoxygenation
NRVM: Neonatal rat ventricular myocytes
